# Effects of Exogenous Melatonin on MAM Induced Lung Injury and Lung Development in Mice Offspring

**Published:** 2020-01

**Authors:** Maryam Azizi, Parichehr Pasbakhsh, Makan Sadr, Tahmineh Mokhtari, Mihan Pourabdollah, Seyed Alireza Nadji, Iraj Ragerdi Kashani

**Affiliations:** 1 Department of Anatomy, School of Medicine, Tehran Medical Sciences Branch, Islamic Azad University, Tehran, Iran,; 2 Department of Anatomical Sciences, Faculty of Medicine, Tehran University of Medical Sciences, Tehran, Iran,; 3 Virology Research Center, National Research Institute of Tuberculosis and Lung Diseases (NRITLD), Shahid Beheshti University of Medical Sciences, Tehran, Iran,; 4 Legal Medicine Research Center, Legal Medicine Organization, Tehran, Iran,; 5 Chronic Respiratory Diseases Research Center, NRITLD, Shahid Beheshti University of Medical Sciences, Tehran, Iran.

**Keywords:** Melatonin, Lung, Methylazoxymethanol, Development, Mice

## Abstract

**Background:**

Melatonin as an antioxidant agent can have an effective role in lung development. In this study, the effect of melatonin administration on lung injury in the neonate mice was assessed.

**Materials and Methods:**

Lung injury was induced by two injections of 15 mg/kg methylazoxymethanol (MAM) on gestational day 15 (E15). Pregnant BALB/c mice were randomly divided into five groups: Control (CO), Melatonin (MEL), Luzindole (Luz), MAM, and MAM+MEL. Melatonin and luzindole were intra-peritoneally injected at a dose of 10 mg/kg (from E15 until delivery). Histopathological changes including: hemorrhage, neutrophils infiltration and fibrosis in the neonate lung were studied by hematoxylin and eosin (H&E) and Masson’s Trichrome staining. Alveolarization and alveolar wall thickness were measured.

**Results:**

In histological examination, hemorrhage, neutrophils infiltration and fibrosis were seen in the MAM and Luz groups; however, these injuries were attenuated in the MAM plus melatonin group. Significant reduction of alveolarization was recorded in the MAM and Luz groups compared to the control group, while the alveolar wall thickness was significantly increased in these groups compared to control group.

**Conclusion:**

Administration of exogenous melatonin in pregnant mice could have a protective effect on the pulmonary development of neonates and could decrease lung injury in neonate mice.

## INTRODUCTION

The process of lung development is very sensitive to environmental toxins. Various studies have shown the toxic effect of these factors on cellular differentiation and function in prenatal and early postnatal periods in children and mice (1). Lung development in mouse begins with growth of a pair of epithelial buds from the foregut on the 9.5 gestational day and later enters the pseudoglandular stage (2). The branching morphogenesis stage begins around 12.5–16.5 of gestational days. In the canalicular stage, proximodistal differentiation occurs between gestational days 16.5–17.5 (3). Around the 17.5 gestational day, lung development reaches the sacculation phase and type I (for gas exchange) and type II (for generation of surfactant) epithelial cells undergo cellular differentiation (4). Finally after birth, lung maturation continues and enters two phases namely: formation of alveolar septum (alveolarization) and microvascular stage (5).

In the early stages of alveoli formation, the alveolar wall develops a double capillary network. In the microvascular maturation stage, the double capillary network comes together, resulting in the merging of the two lumens together and formation of a single network. (6). Decrease in mesenchymal cells and thinning of the connective tissue are two important stages in the alveolar wall maturation (5, 6). Therefore in the postnatal period, a large number of small thin walled alveoli are formed that create a vast area for gas exchange (7). Initially, alveoli formation is increased during postnatal days (P5-P14), gradually decreasing afterwards. Also there is a decrease in the alveolar Septal thickness during P5-P28 (7).

An important point in the lung development is the health issue concerns of the neonate during postnatal period which could be a predisposing factor for the occurrence of pulmonary diseases in the adulthood (8). Studies have shown that adverse perinatal conditions affect the growth and development of lungs that will eventually lead to changes in cardiopulmonary physiology in adults (9). For instance, exposure of neonates to nicotine prevents alveolar growth (10). Also prolonged exposure to hyperoxia results in production of reactive oxygen species (ROS), lung injury and Bronchopulmonary Disorder (11). ROS either directly through oxidative stress or indirectly by induction of apoptosis causes lung injury. In addition, ROS results in release of inflammatory cytokines such as Tumor Necrosis Factor α (TNF)- α and IL-1B. These factors damage the lung parenchyma, impair pulmonary circulation and gas exchange (12).

Methylazoxymethanol (MAM) is a mycotoxin that when administered to rats on day E17 causes structural deficits in the growing offspring (13). MAM is a metabolite of 1,2 dimethylhydrazine (DMH) which induces large amounts of free radicles and affects cellular development and growth (14). Increased cellular levels of free radicles and oxidative stress factors, damages biological molecules including DNA, carbohydrates and proteins (15).

Melatonin, with its anti-oxidant effects, is the main hormone secreted by the pineal gland. The anti-oxidant effect of melatonin is due to:

Upregulating antioxidant enzyme (superoxide dismutase, catalase and glutathione peroxidase), negatively modulating pro-oxidant activity, and scavenging of ROS directly (16).

By inhibiting lipid oxidation, melatonin prevents the oxidative induced injury (17) and also has a protective role in inflammation (18).

The aim of the present study is the evaluation of the protective effects of melatonin on the lung injury induced by MAM and lung development in neonate mice.

## MATERIALS AND METHODS

In this experimental study, 20 adult female Balb/C mice (28–30 gr) were obtained from the Faculty of Veterinary Medicine, Tehran University, and kept under controlled conditions (at a temperature of 22–24°C, with a 12/12-h light/dark cycle, i.e, 6:00 Am-6:00 PM), with food and water available ad libitum.

The mice were coupled with male mice of the same strain overnight, and presence of vaginal plugs the next morning confirmed the pregnancy.

The mice were randomly assigned to five groups: control (Co), Melatonin (MEL), Luzindole (Luz), Methylazoxymethanol (MAM) and MEL+MAM.

Melatonin (Sigma Chemical Co., USA) and Luzindole (Sigma Chemical Co., USA) as a melatonin receptor (MT receptor) antagonist, were administered 10 mg/kg from gestational day 15 (E15) until delivery.

MAM (Wako pure chemical industries, Tokyo, Japan) in normal saline was administered 15 mg/kg twice on E15 at 12:00 PM and 12:00 AM, respectively (19). Control group received normal saline as an equal volume of other drugs, from day E15 until delivery. All drugs were administered by intraperitoneal injection. One month after delivery, six neonates from each group (mix of female and male) were randomly selected and anesthetized with 75 mg/kg ketamine plus 10 mg/kg xylazine intraperitoneally. All mice were perfused intracardially with phosphate buffered saline (PBS) followed by 4% paraformaldehyde, and their lungs were removed and fixed in 10% formalin. The samples were embedded in paraffin and sectioned. The sections were stained with hematoxylin and eosin (H&E) as well as Masson’s trichrome staining for light microscopic assessment.

### Histological assay

The stained sections were assessed by a light microscope (Olympus, CX31, Tokyo, Japan) and images were prepared using a digital camera (Olympus, Japan). At least five or ten fields were randomly selected form different region of the lungs of each mice to study lung development and lung injury.

### Alveolarization assessment

**Figure 1.**
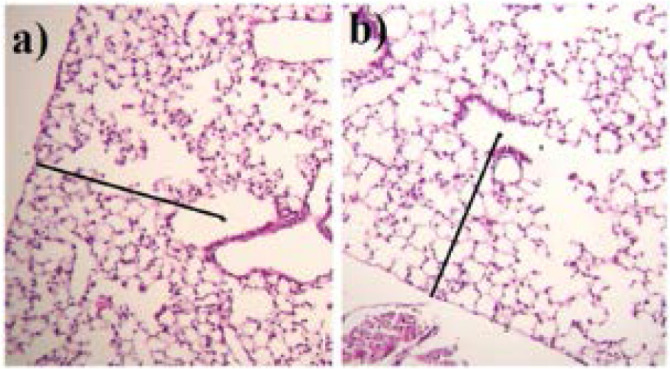
Radial count estimation. a) in the symmetrical respiratory bronchiole. b) in the irregular respiratory bronchiole. H&E staining 40x.

The images were obtained at ×40 and analyzed for radial alveolar count by Emery and Mithal method (20) as follow:

In each field, from the center of respiratory bronchiole a perpendicular line was drawn to the nearest edge of connective tissue septum or pleura, and the alveoli traversed by this line were counted; in symmetrical bronchioles the center of bronchioles was the starting point (Figure 1a), while in the irregular bronchioles midpoint between the proximal part of bronchiole and the first branch point was the starting point (Figure 1b).

### Septal thickness analysis:

To determine the alveolar septal thickness, the images were analyzed by use of image J software (ver. 1.48f, U. S. National institutes of Health; Bethesda 2009).

### Hemorrhage assessment

Presence of red blood cells (RBC) in the fields of images were assessed and scored on a 0–4 scale for hemorrhage (0= no hemorrhage, 2= hemorrhage in parenchyma and small airway, and 4= hemorrhage in large air way) (21).

### Inflammation assessment

Neutrophil infiltration was analyzed by counting neutrophil cells in the fields of images and scored on a 0–4 scale for inflammation (0= no inflammatory cells, 2=1–5 cells/hpf, and 4=>5 inflammatory cells/hpf) (21).

### Assessment of fibrosis

For estimation of percentage of fibrosis in tissue, a number of ten images of random low power fields (x100) of lung were selected from the digital slides in JPG format and fibrosis was assessed using image analysis software in Java (ver. 1.48f, U. S. National institutes of Health; Bethesda 2009).

### Statistical analysis

Data were analyzed using Statistical Package for the Social Sciences software (version 22). The results were presented as mean ± standard SEM. One-way ANOA analysis and post hoc Tukey’s test were done to compare the results among the groups. Statistical significant level was set at P<0.05.

## RESULTS

### Effects of MEL on lung alveolarization in MAM induced pulmonary injury in neonate mice.

**Figure 2.**
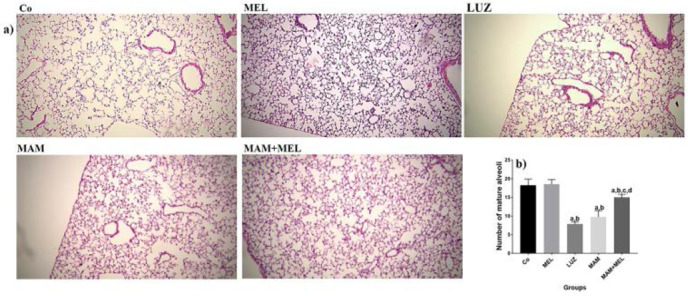
Effects of MEL on lung alveolarization in MAM induced pulmonary injury in neonate mice. a) alveolarization of pulmonary tissue in different groups, H&E staining × 40 magnification. b) Comparing mean alveolarization of pulmonary tissue in different groups. a: p< 0.05 compared to Co group. b: p< 0.05 compared to MEL group, c: p< 0.05 compared to LUZ group, d: p < 0.05 compared to MAM group. Co: Normal saline, MEL: melatonin, LUZ: Luzindole, MAM: Methylazoxymethanole, MAM+MEL Methylazoxymethanole+Melatonin

Mean lung alveolarization was analyzed in different groups (Figure 2-a,b). There was a significant decrease in the lung alveolarization of MAM and LUZ groups compared to Co and MEL groups (P<0.05, Figure 2-a,b). A significant increase in the lung alveolarization of MAM+MEL group was recorded compared to MAM and LUZ group (P<0.05, Figure 2-a,b).

### Effects of MEL on alveolar wall thickness in MAM induced pulmonary injury in neonate mice.

**Figure 3.**
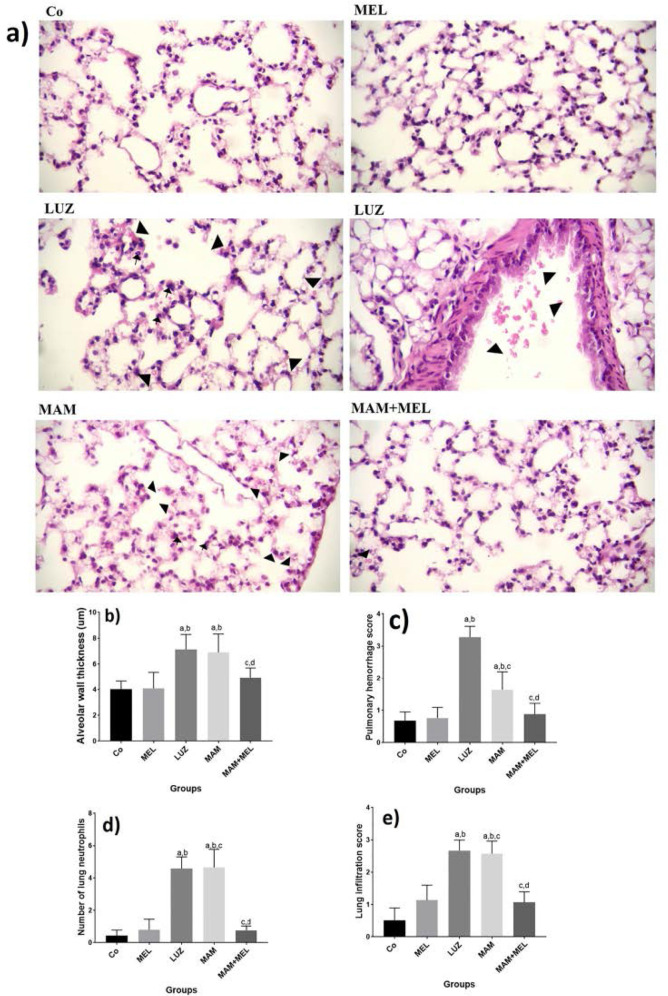
Effects of MEL on alveolar changes in MAM induced pulmonary injury in neonate mice. a) Histopahtologival changes of pulmonary tissue in different groups, H&E staining × 40 magnification. Comparing b) alveolar wall thickness, c) pulmonary hemorrhage score, d) number of lung neutrophils, e) pulmonary infiltration score in different groups. a: p< 0.05 compared to Co group. b: p< 0.05 compared to MEL group, c: p< 0.05 compared to LUZ group, d: p < 0.05 compared to MAM group. Co: Normal saline, MEL: melatonin, LUZ: Luzindole, MAM: Methylazoxymethanole, MAM+MEL Methylazoxymethanole+Melatonin

Mean alveolar wall thickness was analyzed in different groups (Figure 3-a,b). There was a significant increase in the wall thickness of MAM and LUZ groups compared to Co and MEL groups (P<0.05, Figure 3-a,b). A significant decrease in the wall thickness of MAM+MEL group compared to MAM and LUZ groups was observed (P<0.05, Figure 3-a,b).

### Effects of MEL on pulmonary hemorrhage score in MAM induced pulmonary injury in neonate mice.

Mean pulmonary hemorrhage score was compared in different groups (Figure 3-a,c). Significant increase in the pulmonary hemorrhage score of MAM and LUZ groups compared to Co and MEL groups was recorded (P<0.05, Figure 3-a,c). A significant decrease in the pulmonary hemorrhage score of MAM+MEL group compared to MAM and LUZ groups was observed (P<0.05, Figure 3-a,c).

### Effects of MEL on number of lung neutrophils in MAM induced pulmonary injury in neonate mice.

Also, mean number of lung neutrophils was compared in different groups (Figure 3-a,d). Significant increase in the number of lung neutrophils of MAM and LUZ groups compared to Co and MEL groups was recorded (P<0.05, Figure 3-a,d). There was a significant decrease in the number of lung neutrophils of MAM+MEL group compared to MAM and LUZ groups was observed (P<0.05, Figure 3-a,d).

### Effects of MEL on pulmonary infiltration score in MAM induced pulmonary injury in neonate mice.

Mean pulmonary infiltration score was compared in different groups of study (Figure 3-a,e). Significant increase in the pulmonary infiltration score of MAM and LUZ groups compared to Co and MEL groups was recorded (P<0.05, Figure 3-a,e). A significant decrease in the pulmonary infiltration score of MAM+MEL group compared to MAM and LUZ groups was reported (P<0.05, Figure 2-a,e).

### Effects of MEL on pulmonary fibrosis percentage in MAM induced pulmonary injury in neonate mice.

**Figure 4.**
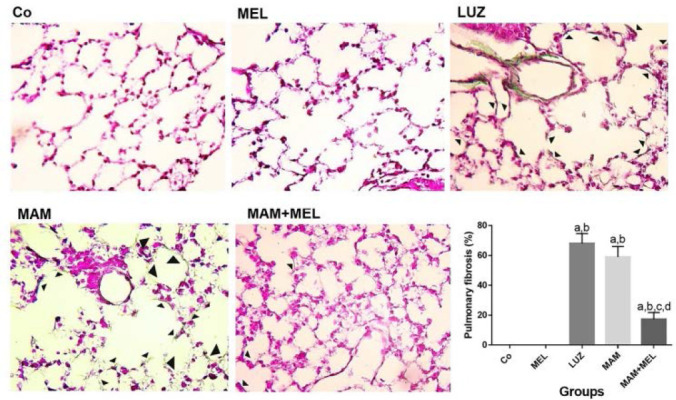
Effects of MEL on mean percentage of pulmonary fibrosis in MAM induced pulmonary injury in neonatal mouse model. a) Histopahtological alteration (arrows) of pulmonary tissue in different groups, Masson’s Trichrome Stain (TRI) staining × 40 magnification. b) Comparing mean percentage of pulmonary fibrosis in different groups. a: p< 0.05 compared to Co group. b: p< 0.05 compared to MEL group, c: p< 0.05 compared to LUZ group, d: p < 0.05 compared to MAM group. Co: Normal saline, MEL: melatonin, LUZ: Luzindole, MAM: Methylazoxymethanole, MAM+MEL Methylazoxymethanole+Melatonin

Mean pulmonary fibrosis percentage was compared in different groups (Figure 4-a,b). There was a significant increase in the pulmonary fibrosis of MAM and LUZ groups compared to Co and MEL groups (P<0.05, Figure 4-a,b). A significant decrease in the pulmonary fibrosis percentage of MAM+MEL group compared to MAM and LUZ groups was recorded (P<0.05, Figure 4-a,b).

## DISCUSSION

In the present study, administration of exogenous melatonin was associated with decreased hemorrhage, neutrophils infiltration, pulmonary fibrosis, and alveolar wall thickness as well as increased alveolarization which ultimately improved lung development in the studied cases.

Administration of MAM to pregnant mice on day E15 of pregnancy, when the number of bronchioles increase (22), causes lung injury and impairs neonatal lung development. Kim et al. announced that MAM has a genotoxic effect in different tissues and increases the production of ROS (23). Also, MAM induces methylation and mutation in DNA; thus, leading to impaired cellular proliferation (24). Ansil et al. reported that administration of 1, 2-dimethylhydrazine, which is metabolized in the liver into MAM, increases the production of malondialdehyde (MDA). MDA is an oxidative agent that is produced as a result of lipid peroxidation and causes oxidative damage (25). MAM leads to oxidative damage in various cellular structures, RNA, DNA, protein and lipids (14).

In a recent study, administration of MAM to pregnant mice increased nitric oxide (NO) as an oxidant agent and decreased anti-oxidant enzymes in cerebral cortex of neonates (26). Studies have shown that lung development is very sensitive to the oxidative damage of oxidants. Eldredge et al. demonstrated that neonate mice exposed to hyperoxia, suffered from lung injury following oxidative stress, that was associated with inflammatory cells activities especially neutrophils (27). Neutrophils and macrophages are important sources of ROS in the lungs (28, 29). Inflammatory cell infiltration by producing proteases, cytokines and ROS affect the lungs and increase the risk of lung injury (30, 31).

Increasing of inflammatory cells activity is associated with increased vascular permeability and hemorrhage (29, 32). In the current study, administration of MAM caused lung injury in the form of hemorrhage, neutrophils infiltration, and pulmonary fibrosis in the offsprings. In the MAM and luzindole groups, there was a significant increase in the amount of hemorrhage and neutrophils infiltration in the lung as compared to the control and Mel groups. However, in the group that received melatonin in addition to MAM (MAM+MEL), neutrophils infiltration and pulmonary fibrosis showed a significant decrease compared to MAM group.

Melatonin as a free radical scavenger has anti-inflammatory and antioxidant characteristics (12). In addition to inhibiting ROS production, it causes activation of anti-oxidant enzymes (33).

Wang et al, reported that administration of melatonin to mouse model of lung ischemia reperfusion injury, suppressed oxidative stress and ameliorated lung injury caused by neutrophils infiltration (12). Zhao and colleagues also evaluated the effect of melatonin administration in pulmonary fibrosis mice model. Their results clearly demonstrated that administration of melatonin significantly decreased bleomycin induced lung fibrosis and luzindole (melatonin receptor inhibitor) suppressed the anti-fibrotic effect of melatonin (34), a point which was in consistent with the present study.

In another research conducted on mice exposed to cigarette smoke and lipopolysaccharide (LPS), lung injury was observed as fibrosis, increased ROS production and neutrophil cell count. While in mice treated with melatonin, significant decrease in ROS production, neutrophil cell count and fibrosis was reported. This finding confirmed that melatonin through its anti-oxidant and anti-inflammatory effect suppressed inflammatory response and fibrosis in lung tissues of the samples (35). This finding was also in consistent with the results of the present study. Tahamtan et al. reported lung injury in the form of fibrosis, increased RBC and neutrophil cell count in rats exposed to radiation, in which lung injury was reversed with melatonin administration (36).

In the present research both lung injury and lung development of the samples was studied. Alveolarization was significantly decreased in the MAM and Luz groups as compared to Control and Mel groups, while it was significantly increased in the melatonin group in comparison to MAM group.

The present study was in consistent with the study conducted by Yildiz et al. in which melatonin was administered to neonatal rats receiving maternal nicotine; results showed significant increase in the number of alveoli as compared to the group that did not receive melatonin treatment (17). During growth, capillary network development is essential in the process of gas exchange and during its development alveolar septal wall thickness decreases (37). In the MAM and Luz groups, alveolar septal wall thickness showed significant increase in comparison to Mel and Control groups. However, in MAM+MEL group alveolar septal wall thickness decreased considerably as compared to the MAM group. Xu et al. demonstrated that neonatal rats when exposed to hyperoxia, developed lung fibrosis and increased alveolar septal wall thickness (38).

Oxidative stress condition leads to neutrophil cells infiltration in lungs and subsequent alveolarization disorder. The present study is also in consistent with Ryan et al study in which suppression of inflammatory mediators or their receptors could improve lung development especially the alveoli. Thus, increase in the number of inflammatory cells or even inflammation is associated with impaired lung development and growth, increased alveolar septal wall thickness, and destruction of alveoli is accompanied by lung fibrosis (31).

## CONCLUSION

Exogenous melatonin had protective effects on lung injury by decreasing of fibrosis, hemorrhage and neutrophils infiltration. In addition, administration of melatonin could improve the lung development by decreasing of alveolar wall thickness as well as increasing of alveolarization in neonate mice.

## References

[1] Cao J: Early life exposure to toxic environments: effects on lung and immune cell development in mice and men: University of Groningen, 2016.

[2] Morrisey EE Hogan BL. Preparing for the first breath: genetic and cellular mechanisms in lung development. Dev Cell 2010;18(1):8–23. 20152174 10.1016/j.devcel.2009.12.010 PMC3736813

[3] Snoeck HW. Modeling human lung development and disease using pluripotent stem cells. Development 2015;142(1):13–6. 25516965 10.1242/dev.115469

[4] Wang X Wang Y Snitow ME Stewart KM Li S Lu M Expression of histone deacetylase 3 instructs alveolar type I cell differentiation by regulating a Wnt signaling niche in the lung. Dev Biol 2016;414(2):161–9. 27141870 10.1016/j.ydbio.2016.04.023 PMC4975046

[5] Kugler MC Joyner AL Loomis CA Munger JS. Sonic hedgehog signaling in the lung. From development to disease. Am J Respir Cell Mol Biol 2015;52(1):1–13. 25068457 10.1165/rcmb.2014-0132TR PMC4370254

[6] Burri PH. Structural aspects of postnatal lung development - alveolar formation and growth. Biol Neonate 2006;89(4):313–22. 16770071 10.1159/000092868

[7] Pozarska A Rodriguez-Castillo JA Surate Solaligue DE Ntokou A Rath P Mizikova I Stereological monitoring of mouse lung alveolarization from the early postnatal period to adulthood. Am J Physiol Lung Cell Mol Physiol 2017;312(6):L882–L95. 28314804 10.1152/ajplung.00492.2016

[8] Corrin B Nicholson AG: Pathology of the Lungs E-Book: Expert Consult: Online and Print: Elsevier Health Sciences, 2011: pp.

[9] Ali M Heyob KM Velten M Tipple TE Rogers LK. DHA suppresses chronic apoptosis in the lung caused by perinatal inflammation. Am J Physiol Lung Cell Mol Physiol 2015;309(5):L441–8. 26138643 10.1152/ajplung.00137.2015 PMC4556930

[10] McGrath-Morrow SA Hayashi M Aherrera A Lopez A Malinina A Collaco JM The effects of electronic cigarette emissions on systemic cotinine levels, weight and postnatal lung growth in neonatal mice. PLoS One 2015;10(2):e0118344. 25706869 10.1371/journal.pone.0118344 PMC4338219

[11] Tokuriki S Igarashi A Okuno T Ohta G Naiki H Ohshima Y. Treatment with Geranylgeranylacetone Induces Heat Shock Protein 70 and Attenuates Neonatal Hyperoxic Lung Injury in a Model of Bronchopulmonary Dysplasia. Lung 2017;195(4):469–76. 28447205 10.1007/s00408-017-0007-4 PMC5522658

[12] Wang ML Wei CH Wang WD Wang JS Zhang J Wang JJ. Melatonin attenuates lung ischaemia-reperfusion injury via inhibition of oxidative stress and inflammation. Interact Cardiovasc Thorac Surg 2018;26(5):761–7. 29346581 10.1093/icvts/ivx440

[13] Ratajczak P Kus K Murawiecka P Slodzinska I Giermaziak W Nowakowska E. Biochemical and cognitive impairments observed in animal models of schizophrenia induced by prenatal stress paradigm or methylazoxymethanol acetate administration. Acta Neurobiol Exp (Wars) 2015;75(3):314–25. 26581387

[14] Syed U Ganapasam S. Beneficial influence of ellagic acid on biochemical indexes associated with experimentally induced colon carcinogenesis. J Cancer Res Ther 2017;13(1):62–8. 28508835 10.4103/0973-1482.172715

[15] Jaya Gupta AGaAKG. The Flavonoids Ameliorates: Protective Mechanisms in Neurodegenerative Diseases. Int J Curr Res Chem Pharm Sci 2017;4(1): 1–7.

[16] Hardeland R. Antioxidative protection by melatonin: multiplicity of mechanisms from radical detoxification to radical avoidance. Endocrine 2005;27(2):119–30. 16217125 10.1385/endo:27:2:119

[17] Yildiz A Vardi N Karaaslan MG Ates B Taslidere E Esrefoglu M. The protective effect of melatonin in lungs of newborn rats exposed to maternal nicotine. Biotech Histochem 2018;93(6):442–52. 29701082 10.1080/10520295.2018.1453548

[18] Zhang L Zhang F He D Xu D Zhong Z Shen J. Melatonin attenuates phosgene-induced acute lung injury via the upregulation Wnt/β-catenin pathway. International Journal of Clinical and Experimental Pathology. 2017;10(11):11281–7. 31966482 PMC6965881

[19] Marchi N Guiso G Caccia S Rizzi M Gagliardi B Noe F Determinants of drug brain uptake in a rat model of seizure-associated malformations of cortical development. Neurobiol Dis 2006;24(3):429–42. 17027274 10.1016/j.nbd.2006.07.019

[20] Datta A Kim GA Taylor JM Gugino SF Farrow KN Schumacker PT Mouse lung development and NOX1 induction during hyperoxia are developmentally regulated and mitochondrial ROS dependent. Am J Physiol Lung Cell Mol Physiol 2015;309(4):L369–77. 26092998 10.1152/ajplung.00176.2014 PMC4587628

[21] Polglase GR Barbuto J Allison BJ Yawno T Sutherland AE Malhotra A Effects of antenatal melatonin therapy on lung structure in growth-restricted newborn lambs. J Appl Physiol (1985) 2017; 123:1195–1203. 28819007 10.1152/japplphysiol.00783.2016

[22] Snyder JM. Versican Expression During Embryonic Development in the Mouse 2014.

[23] Kim DH Sung B Chung HY Kim ND. Modulation of Colitis-associated Colon Tumorigenesis by Baicalein and Betaine. J Cancer Prev 2014;19(3):153–60. 25337584 10.15430/JCP.2014.19.3.153 PMC4189507

[24] Jikihara H Qi G Nozoe K Hirokawa M Sato H Sugihara Y Aged garlic extract inhibits 1,2-dimethylhydrazine-induced colon tumor development by suppressing cell proliferation. Oncol Rep 2015;33(3):1131–40. 25573280 10.3892/or.2014.3705

[25] Ansil P Jazaira V Prabha S Nitha A Latha M. Amorphophallus campanulatus (roxb.) blume. tuber ameliorates hepatic oxidative stress during colon carcinogenesis induced by 1, 2 dimethylhydrazine. International Journal of Pharmacy and Pharmaceutical Sciences 2013;5(1):366–71.

[26] Azizi M Pasbakhsh P Nadji SA Pourabdollah M Mokhtari T Sadr M Therapeutic effect of perinatal exogenous melatonin on behavioral and histopathological changes and antioxidative enzymes in neonate mouse model of cortical malformation. Int J Dev Neurosci 2018; 68:1–9. 29605566 10.1016/j.ijdevneu.2018.03.008

[27] Eldredge LC Treuting PM Manicone AM Ziegler SF Parks WC McGuire JK. CD11b(+) Mononuclear Cells Mitigate Hyperoxia-Induced Lung Injury in Neonatal Mice. Am J Respir Cell Mol Biol 2016;54(2):273–83. 26192732 10.1165/rcmb.2014-0395OC PMC4821040

[28] Rossi A Serraino I Dugo P Di Paola R Mondello L Genovese T Protective effects of anthocyanins from blackberry in a rat model of acute lung inflammation. Free Radic Res 2003;37(8):891–900. 14567449 10.1080/1071576031000112690

[29] Perrone S Tataranno ML Buonocore G. Oxidative stress and bronchopulmonary dysplasia. J Clin Neonatol 2012;1(3):109–14. 24027702 10.4103/2249-4847.101683 PMC3762019

[30] Xia H Ren X Bolte CS Ustiyan V Zhang Y Shah TA Foxm1 regulates resolution of hyperoxic lung injury in newborns. Am J Respir Cell Mol Biol 2015;52(5):611–21. 25275225 10.1165/rcmb.2014-0091OC PMC4491137

[31] Ryan RM Ahmed Q Lakshminrusimha S. Inflammatory mediators in the immunobiology of bronchopulmonary dysplasia. Clin Rev Allergy Immunol 2008;34(2):174–90. 18330726 10.1007/s12016-007-8031-4

[32] Cai Y Bolte C Le T Goda C Xu Y Kalin TV FOXF1 maintains endothelial barrier function and prevents edema after lung injury. Sci Signal 2016;9(424):ra40. 27095594 10.1126/scisignal.aad1899

[33] Goc Z Szaroma W Kapusta E Dziubek K. Protective effects of melatonin on the activity of SOD, CAT, GSH-Px and GSH content in organs of mice after administration of SNP. Chin J Physiol 2017;60(1):1–10. 28052641 10.4077/CJP.2017.BAF435

[34] Zhao X Sun J Su W Shan H Zhang B Wang Y Melatonin Protects against Lung Fibrosis by Regulating the Hippo/YAP Pathway. Int J Mol Sci 2018;19(4). 10.3390/ijms19041118 PMC5979295 29642520

[35] Shin NR Park JW Lee IC Ko JW Park SH Kim JS Melatonin suppresses fibrotic responses induced by cigarette smoke via downregulation of TGF-beta1. Oncotarget 2017;8(56):95692–703. 29221159 10.18632/oncotarget.21680 PMC5707053

[36] Tahamtan R Shabestani Monfared A Tahamtani Y Tavassoli A Akmali M Mosleh-Shirazi MA Radioprotective effect of melatonin on radiation-induced lung injury and lipid peroxidation in rats. Cell J 2015;17(1):111–20. 25870840 10.22074/cellj.2015.517 PMC4393658

[37] Tibboel J Groenman FA Selvaratnam J Wang J Tseu I Huang Z Hypoxia-inducible factor-1 stimulates postnatal lung development but does not prevent O2-induced alveolar injury. Am J Respir Cell Mol Biol 2015;52(4):448–58. 25180700 10.1165/rcmb.2014-0037OC

[38] Xu W Zhao Y Zhang B Xu B Yang Y Wang Y Resveratrol attenuates hyperoxia-induced oxidative stress, inflammation and fibrosis and suppresses Wnt/beta-catenin signalling in lungs of neonatal rats. Clin Exp Pharmacol Physiol 2015;42(10):1075–83. 26174235 10.1111/1440-1681.12459

